# Wetting Transition from Wenzel to Cassie States: Thermodynamic Analysis

**DOI:** 10.3390/ma18030543

**Published:** 2025-01-24

**Authors:** Qiang Sun, Yan-Nan Chen, Yu-Zhen Liu

**Affiliations:** Key Laboratory of Orogenic Belts and Crustal Evolution, Ministry of Education, The School of Earth and Space Sciences, Peking University, Beijing 100871, China; 2301210129@stu.pku.edu.cn (Y.-N.C.); liuyuzhen@stu.pku.edu.cn (Y.-Z.L.)

**Keywords:** superhydrophobicity, surface roughness, Wenzel–Cassie transition, *W*_Roughness_, hydrogen bonding

## Abstract

Superhydrophobicity is closely linked to the chemical composition and geometric characteristics of surface roughness. Building on our structural studies on water and air–water interfaces, this work aims to elucidate the mechanism underlying the wetting transition from the Wenzel to the Cassie state on a hydrophobic surface. In the Wenzel state, the grooves are filled with water, meaning that the surface roughness becomes embedded in the liquid. To evaluate the effects of surface roughness on water structure, a wetting parameter (*W*_Roughness_) is proposed, which is closely related to the geometric characteristics of roughness, such as pillar size, width, and height. During the wetting transition from Wenzel to Cassie states, the critical wetting parameter (*W*_Roughness,c_) may be expected, which corresponds to the critical pillar size (*a*_c_), width (*w*_c_), and height (*h*_c_). The Cassie state is expected when the *W*_Roughness_ is less than *W*_Roughness,c_ (<*W*_Roughness,c_), which can be achieved by altering the geometric characteristics of the roughness, such as increasing pillar size (>*a*_c_), decreasing width (<*w*_c_), or increasing height (>*h*_c_). Additionally, molecular dynamic (MD) simulations are conducted to demonstrate the effects of surface roughness on superhydrophobicity.

## 1. Introduction

Wettability, which describes how liquids interact with a solid surface, is a crucial property from both fundamental and practical perspectives. Recently, bioinspired surfaces [1,2,3] exhibiting superhydrophobicity—an extreme form of surface wettability—have garnered significant attention. Superhydrophobicity plays a critical role in various applications, including self-cleaning materials [4,5], corrosion inhibition layers on metal surfaces [6,7], anti-icing surfaces [8,9], anti-flashover coatings [10,11], and oil/water separation membranes and meshes [12,13,14,15]. It is important to investigate the mechanism behind superhydrophobic surfaces.

**Figure 1:**
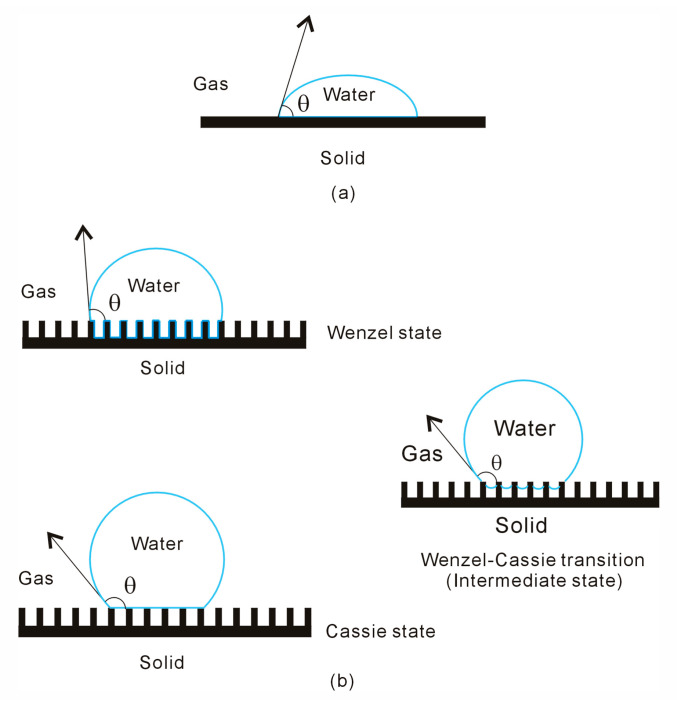
(**a**) The wettability of water droplets on solid surface. (**b**) The intermediate state is expected during the wetting transition from Wenzel to Cassie states.

The fundamental law governing the equilibrium shape of a liquid drop on a surface was formulated by Thomas Young [16] in 1805. Young’s equation arises from the thermodynamic equilibrium of free energy at the solid–liquid–vapor interfaces [17,18]. According to Young’s equation [16], when a liquid droplet is placed on a solid surface, equilibrium is achieved at a specific angle known as the static contact angle (CA, *θ*) (Figure 1),

(1) $$\cos\theta =\frac{\gamma_{\mathrm{SG}}-\gamma_{\mathrm{SL}}}{\gamma_{\mathrm{LG}}}$$

where *γ*_SL_, *γ*_SG_ and *γ*_LG_ represent the surface energies of the solid–liquid, solid–air and liquid–air interfaces, respectively.

A surface is considered superhydrophobic when the CA exceeds 150° and the contact angle hysteresis (CAH) is less than 10°. CAH, defined as the difference between the advancing contact angle (*θ*_adv_) and the receding contact angle (*θ*_rec_), effectively characterizes the adhesion of drops on solid surfaces and is a crucial parameter for various engineering applications of superhydrophobic surfaces [19,20,21]. The hydrophobicity of solid surfaces is influenced by both their chemical composition and their micro- or nanoscale geometry. Chemical modifications alone, such as fluoropolymeric coatings or silane layers [22], typically achieve water CA up to 120° but no higher. To achieve extreme CA near 180°, superhydrophobicity is enhanced by surface roughness, a phenomenon often referred to as the Lotus effect [23].

In general, the Wenzel model [24,25] and the Cassie–Baxter model [26] are applied to understand the effect of surface roughness on the apparent CA (θ*) of liquid drops. The Wenzel model, as an extension of Young’s equation, applies the concept of CA to rough, chemically homogeneous surfaces. According to Wenzel’s equation, the θ* can be expressed as follows:

(2) $$\cos\theta^{*}=r\cdot \cos\theta$$

where the roughness factor (*r*) is defined as the ratio of the actual area of a rough surface to its geometric projected area. In the Cassie model [26], it is assumed that the liquid drop cannot penetrate the cavities on the rough surface, trapping air in these cavities and forming a composite interface. Cassie and Baxter [26] proposed the following equation for this model:

(3) $$\cos\theta^{*}=f_{\mathrm{SL}}\cos\theta_{1}+f_{\mathrm{LG}}\cos\theta_{2}$$

in which *θ*_1_ and *θ*_2_ represent the CAs of the liquid in contact with the solid surface and air, *f*_SL_ is the area fraction of the liquid droplet in contact with the solid surface, and *f*_LG_ is the area fraction in contact with air trapped in the pores of the rough surface, *f*_SL_ + *f*_LG_ = 1. In this composite interface, the air portions of the surface are considered perfectly non-wetting, with *θ*_1_ = 0 and *θ*_2_ = 180°. Therefore, the equation is expressed as:

(4) $$\cos\theta^{*}=f_{\mathrm{SL}}\left(\cos\theta +1\right)-1$$

However, for surfaces with irregular micronanostructures, it is difficult to interpret the wettability of the surface directly using the classical Wenzel model and the Cassie model. Onda et al. [27,28] introduced the fractal theory to investigate the complex surface with fractal structures and obtain a fractal-wetting model. In Davis et al.’s [29] work, they showed no clear correlation between the static CA and the fractal dimensions.

In the Wenzel state, the adhesion between the water droplet and the textured surface is stronger than in the Cassie state [30]. As a result, the Cassie state is often preferred in many practical applications. It is crucial to understand the wetting transition from the Wenzel to the Cassie state. As CA increases, the critical threshold angle (*θ*_c_) between two regimes can be determined by equating the Wenzel and Cassie equations [31], which is given as:

(5) $$\cos\theta_{c}=\frac{f_{\mathrm{SL}}-1}{r-f_{\mathrm{SL}}}$$

This suggests that the transition from the Wenzel to the Cassie state can be influenced by the geometric characteristics of surface roughness.

Recently, carbon nanotubes and graphene have garnered significant attention [32]. To investigate the relationship between surface roughness and the Wenzel–Cassie transition, many works [33,34,35,36,37,38] have been conducted. Molecular dynamics (MD) simulations by Lundgren et al. [34] explored variations in the CA concerning pillar height, observing a transition between the Wenzel and Cassie–Baxter regimes with changes in pillar height. Niu and Tang [35] investigated the droplet state on surfaces with varying roughness, finding that it depended on both pillar height and the area ratio occupied by the pillars. Thus, a close relationship between the Wenzel–Cassie transition and the geometric characteristics of surface roughness is expected. Understanding this relationship is crucial for effectively designing superhydrophobic surfaces.

Superhydrophobicity is influenced by the chemical composition [39,40,41] and geometric characteristics of surface roughness. This work aims to elucidate the mechanism of Wenzel–Cassie transition on hydrophobic surfaces. Based on the structural studies [42,43,44] on water and air–water interface, surface roughness mainly affects the structure of the topmost water layer at the roughness–water interface (interfacial water). To evaluate the effects of surface roughness on water structure, we propose a wetting parameter (*W*_Roughness_). During the wetting transition from Wenzel to Cassie states, a critical wetting parameter (*W*_Roughness,c_) is found, which is due to the transition between interfacial and bulk water. Additionally, *W*_Roughness,c_ also corresponds to the critical pillar size (*a*_c_), width (*w*_c_), and height (*h*_c_). The Cassie state is expected when the *W*_Roughness_ being less than *W*_Roughness,c_ (<*W*_Roughness,c_), which is achieved by increasing pillar size (>*a*_c_), decreasing width (<*w*_c_), or increasing height (>*h*_c_). Furthermore, MD simulations are also conducted to demonstrate the effects of surface roughness on superhydrophobicity.

## 2. Methods

### 2.1. MD Simulations

**Figure 2:**
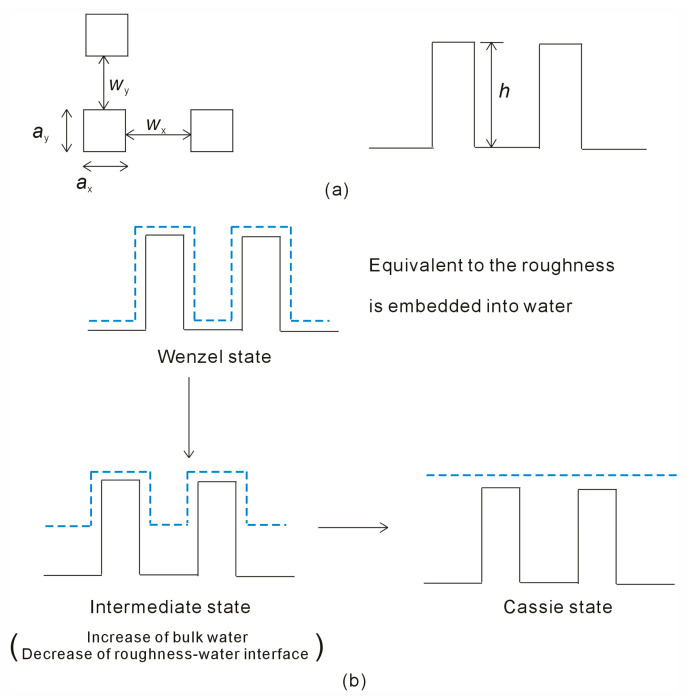
(**a**) The square roughness may be characterized by the roughness size, the width between roughnesses, and roughness height. (**b**) As a water droplet is placed on the surface roughness, the roughness mainly affects the structure of interfacial water. The Wenzel–Cassie transition may be associated with a structural rearrangement of the liquid water. Interfacial water is shown in dashed line.

To explore the relationship between superhydrophobicity and surface roughness, MD simulations are conducted using the GROningen MAchine for Chemical Simulations (GROMACS) package (version: Gromacs 2019.6) [45,46]. In this investigation, square pillars are employed to model surface roughness, characterized by parameters such as the pillar side lengths in the x and y axes (*a*_x_, *a*_y_), the groove widths between pillars in the x and y axes (*w*_x_, *w*_y_), and the pillar height (*h*) (Figure 2 and Table 1). The effects of surface roughness on superhydrophobicity are examined by varying geometric characteristics, including side length, separations, or pillar height (Table 1).

In the MD simulations, a cubic water box of dimensions 60 Å × 60 Å × 60 Å is initially positioned on the graphite substrate with various surface roughness (Table 1). Upon reaching thermodynamic equilibrium, the liquid phase may infiltrate into the grooves of the surface roughness (resulting in the Wenzel state) or rest atop the peaks of the surface roughness (yielding the Cassie state). Additionally, to examine the correlation between superhydrophobicity and drop size, cubic liquids of other dimensions are also employed, including 30 Å × 30 Å × 30 Å, 40 Å × 40 Å × 40 Å, and 50 Å × 50 Å × 50 Å.

During the simulations, the Optimized Potential for Liquid Simulations-All Atom (OPLS-AA) force field was utilized to model the interactions among carbon atoms in graphite. Various water models have been developed for simulating water molecules. For this study, the extended simple point charge (SPC/E) model was employed for the water molecules. Simulations were conducted in the canonical ensemble (NVT). The temperature was maintained at 300 K using Nose-Hoover thermostat dynamics. The simulated box dimensions were 200 Å × 200 Å × 200 Å, with the graphite substrate held fixed. Additionally, periodic boundary conditions were applied in all three directions. Lennard–Jones interactions were truncated at 1.0 nm, and the particle mesh Ewald method was employed to compute long-range electrostatic forces. Each simulation ran for 1 ns, with a time step of 2 fs.

### 2.2. CA Measurements

The CA can be defined as the angle between the substrate and a tangential line on the surface of the droplet, where the tangential line intersects a three-phase contact point and its plane, when projected onto the substrate, passes through the center of the droplet. CA measurement techniques are generally classified into two types: direct and indirect methods. From the MD simulations, equilibrated regular droplet configurations, near sphere shape with radius about 40 Å, were extracted from the trajectories. The CAs between the water droplet and the substrate were then determined using the public domain software ImageJ (version: 1.54g) [47] with a contact angle plug-in.

For each measurement, the user must manually select two points to define the baseline and three points along the droplet profile. Once the points are selected, the software fits the droplet profile and calculates the contact angle using either the sphere approximation or the ellipse approximation [48]. In this study, the sphere approximation was used. Additionally, each configuration was measured three times, and the calculated contact angle had an approximate error of 3°.

### 2.3. Hydrogen Bonding

Because hydrogen bondings are stronger than van der Waals forces, water may play a critical role in the wetting transition from Wenzel to Cassie states. Therefore, it is important to investigate the changes in hydrogen bondings in water during the Wenzel–Cassie wetting transition. In the work, the geometrical definition of hydrogen bonding is utilized to determine the hydrogen bondings in liquid water [49]. According to the geometrical definition, hydrogen bonding is considered to exist between two neighboring water molecules if the Oxygen–Oxygen distance (*r*_OO_) is smaller than 3.5 Å, and ∠OOH angle between two water molecules is less than 30°, respectively. In this study, the hydrogen bonding of water was calculated using the Visual Molecular Dynamics (VMD) program (version: vmd 1.9.3) [50].

## 3. Results and Discussion

### 3.1. Thermodynamic Analysis of Wenzel–Cassie Transition

It is widely recognized that superhydrophobicity is closely related to the surface roughness of solid surfaces. Due to the surface roughness, this increases the CA between the solid surface and a water droplet (Figure 1). During the wetting transition from the Wenzel to Cassie states, the contact area between the liquid and solid surfaces decreases. This suggests that the Wenzel–Cassie transition may be associated with changes in the interactions between water and surface roughness.

In the Wenzel state, the grooves are completely filled with liquid water (Figure 2). After considering the volume of the surface roughness, this is equivalent to the surface roughness being embedded into the water in the Wenzel state. During the Wenzel–Cassie transition, the contact area between the water and surface roughness decreases. Of course, this is also accompanied by an increase in bulk water. This suggests that the Wenzel–Cassie transition may be associated with a structural rearrangement of the liquid water (Figure 2). Therefore, it is necessary to investigate the structure of liquid water, and the effects of surface roughness on water structure.

Water is often considered an anomalous liquid due to the formation of hydrogen bonds between neighboring water molecules. Numerous experimental and theoretical studies have been conducted to understand the structure of liquid water, leading to the development of various structural models. These models are broadly categorized into two groups: (a) mixture models and (b) continuum (or distorted-hydrogen bonding) models [51,52]. The mixture model suggests that ambient water simultaneously possesses two distinct types of structures. In contrast, the continuum structural model proposes that water is composed of a random, three-dimensional network of hydrogen bonds, characterized by a wide distribution of O-H∙∙∙O hydrogen bond angles and distances. However, unlike the mixture models, the hydrogen-bonded networks in the continuum model cannot be separated into distinct molecular species.

Due to the formation of hydrogen bonds between neighboring water molecules, this causes OH vibrations to shift to lower wavenumbers. Therefore, OH vibrations may be sensitive to hydrogen bonding and are widely utilized to probe the structure of water. In our Raman spectroscopic studies [42,43], it was observed that, as three-dimensional hydrogen bonding arrangements emerge, OH vibrations primarily depend on the hydrogen bonding within the first shell of a water molecule (local hydrogen bonding). The influence of hydrogen bonding beyond the first shell on OH vibrations may be weak. Consequently, different OH vibrations can be reasonably attributed to OH vibrations engaged in various local hydrogen-bonded networks of a water molecule.

Under ambient conditions, the Raman OH stretching bands of water can be deconvoluted into five sub-bands, each corresponding to OH vibrations involved in different types of local hydrogen bondings. These include double donor-double acceptor (DDAA, tetrahedral hydrogen bonding), double donor-single acceptor (DDA), single donor-double acceptor (DAA), and single donor-single acceptor (DA) hydrogen bonds. Unlike the mixture and continuum models, this indicates that a water molecule interacts with neighboring water molecules through various local hydrogen-bonded networks [42,43]. Additionally, the hydrogen-bonded networks in water can be influenced by factors such as temperature, pressure, dissolved salts, and confined environments, which may lead to the networks being rearranged in response to these changes.

In the Wenzel state, the liquid phase can infiltrate into the grooves of the solid surface. This is equivalent to the surface roughness becoming embedded in the water. Due to the appearance of surface roughness–water interface, this inevitably influences the structure of liquid water. Because OH vibration is closely related to the local hydrogen bonding of water, surface roughness primarily affects the structure of interfacial water. Indeed, numerous studies on the structure and dynamics of water surrounding ions, conducted through techniques such as neutron and X-ray diffraction [53], X-ray absorption spectroscopy [54], femtosecond time-resolved infrared (fs-IR) vibrational spectroscopy [55,56], and optical Kerr-effect spectroscopy [57], support this notion. These suggest that the effects of dissolved solutes on water structure are predominantly confined to the first solvation shell.

Indeed, vibrational sum frequency generation (SFG) spectroscopy is a powerful method for probing the molecular-level details of surfaces and interfaces. Numerous experimental SFG studies have been conducted to unravel the structure of the air–water interface [58,59,60,61]. Additionally, the development of phase-sensitive sum-frequency generation (PS-SFG) spectroscopy by Shen et al. [62,63] has offered new insights. Through PS-SFG measurements, the sign of the imaginary part of χ(2) (Imχ(2)) indicates that the water molecular dipole direction can be directly determined. Based on our SFG study of the air–water interface [44], it is impossible to form DDAA hydrogen bonding in the interfacial water. Therefore, an obvious structural distinction may be expected across the interface.

Based on the structural studies on water and air–water interface [42,43,44], it is found that DA hydrogen bondings tend to form in interfacial water. Additionally, the loss of DDAA hydrogen bonding is associated with the formation of the solute–water interface. Therefore, when the ratio of the interfacial water layer to volume is determined, this may be used to calculate the Gibbs free energy between the solute and water (Δ*G*_Solute-water_)

(6) $$\Delta G_{\mathrm{Solute}-\mathrm{water}}=R_{\mathrm{Interfacial}\;\mathrm{water}/\mathrm{Bulk}\;\mathrm{water}}\cdot \Delta G_{\mathrm{DDAA}}\cdot n_{\mathrm{HB}}$$

where *R*_Interfacial water/Bulk water_ represents the ratio of interfacial to bulk water, Δ*G*_DDAA_ stands for the Gibbs energy of DDAA hydrogen bondings, and *n*_HB_ denotes the average number of hydrogen bondings per water molecule. For DDAA hydrogen bondings of water, *n*_HB_ equals 2.

For a square roughness, the side length is represented by *a*_x_ and *a*_y_ in the x and y axes, the separation between neighboring pillars is denoted as *w*_x_ and *w*_y_, and the pillar height is *h* (Figure 2). Based on Equation (6), the Gibbs free energy of interfacial water, associated with the roughness of the solid surface (Δ*G*_Roughness-water_), can be expressed as

(7) $$\Delta G_{\mathrm{Roughness}-\mathrm{water}}=2\cdot \Delta G_{\mathrm{DDAA}}\cdot \frac{\left(\left(a_{x}+w_{x}\right)\left(a_{y}+w_{y}\right)+\left(2a_{x}+2a_{y}\right)h\right)/\left(\pi r_{H2O}^{2}\right)}{\left(a_{x}a_{y}h\right)/\left(4\pi r_{H2O}^{3}/3\right)}$$

in which *r*_H2O_ is the radius of a water molecule.

The surface roughness mainly affects the structure of interfacial water. When a droplet is placed on a solid surface, it can be divided into interfacial and bulk water. During the wetting transition from the Wenzel to Cassie states, there is a reduction in the contact area between the surface roughness and the water, which is associated with a decrease in interfacial water and an increase in bulk water (Figure 2). Consequently, there exists a close relationship between interfacial and bulk water during the Wenzel–Cassie wetting transition, attributable to the effects of surface roughness on water structure. When the Wenzel–Cassie transition occurs, the following equation is expected

(8) $$\Delta G_{\mathrm{Roughness}-\mathrm{water}}=\Delta G_{\mathrm{Water}-\mathrm{water}}$$

In our previous works [64,65], hydration-free energy is derived and applied to understand the mechanism behind hydrophobic effects. Hydration-free energy is the sum of ∆*G*_Water-water_ and ∆*G*_Solute-water_, it may be dominated by ∆*G*_Water-water_ or ∆*G*_Solute-water_, depending on the solute size. As in Equation (8), a transition is expected when Δ*G*_Water-water_ equals Δ*G*_Solute-water_, and critical radius (*R*_c_) is expected for the solute. With reference to *R*_c_, it is divided into initial and hydrophobic solvation processes, and various dissolved behaviors are expected for solutes in different processes, such as dispersed or aggregated distributions in water. Therefore, hydrophobic effects are reasonably ascribed to the structural competition between interfacial and bulk water.

In combination with our studies [64,65] on hydrophobic interactions, the Wenzel–Cassie transition could be related to the structural competition between interfacial and bulk water. Equation (8) may be employed to explore the thermodynamic conditions during the wetting transition from Wenzel to Cassie states. This analysis is based on the following assumptions: (I) the size of the water droplet is significantly larger than that of the surface roughness, and (II) it is reasonable to disregard the influence of gravity on wettability. Based on Equations (7) and (8), the following equation is derived

(9) $$\underset{W_{\mathrm{Roughness}}}{\underset{︸}{\frac{1}{h}\cdot \frac{\left(a_{x}+w_{x}\right)\left(a_{y}+w_{y}\right)}{a_{x}a_{y}}+\frac{2\left(a_{x}+a_{y}\right)}{a_{x}a_{y}}}}=\underset{W_{\mathrm{Water}}}{\underset{︸}{\frac{3}{8r_{H2O}}\cdot \frac{\Delta G_{\mathrm{Water}-\mathrm{water}}}{\Delta G_{\mathrm{DDAA}}}}}$$

in which *W* is termed as the wetting parameter, *W*_Roughness_ and *W*_Water_ are the corresponding parameters, respectively, related to surface roughness and liquid water.

In the Wenzel state, the water droplet fully wets the solid rough surface, with the surface roughness being completely covered by interfacial water. In the Cassie state, the droplet rests on the peaks of the rough surface geometry, with the liquid phase not penetrating the surface roughness (Figure 2). In general, a water droplet partially wets the peaks of a superhydrophobic surface, with air trapped in the grooves of the substrate. During the wetting transition from the Wenzel to Cassie states, an intermediate state typically occurs [66,67], which can be described by Equation (9).

According to Equation (9), *W*_Water_ is directly influenced by the thermodynamic properties of liquid water. At 293 K and 0.1 MPa, the Δ*G*_Water-water_ is −6276 J/mol [68]. Additionally, the average volume of a water molecule is approximately 3 × 10^−29^ m^3^ under ambient conditions. Considering the water molecule as a sphere, its corresponding diameter is 3.8 Å, and *r*_H2O_ is 1.9 Å. Furthermore, the Δ*G*_DDAA_ is calculated to be −2.66 kJ/mol at 293 K and 0.1 MPa [65]. At 293 K and 0.1 MPa, *W*_Water_ is determined to be 0.47.

From the above discussion, the critical *W*_Roughness_ (*W*_Roughness,c_) may be expected during the wetting transition from Wenzel to Cassie states. Based on Equation (9), *W*_Roughness,c_ can be determined. Additionally, *W*_Roughness,c_ may be characterized by the critical geometric characteristics of surface roughness, such as *a*_c_, *w*_c_, and *h*_c_. With reference to *W*_Roughness,c_, it may be divided into the Wenzel or Cassie states, and described as follows

(10) $$\begin{array}{l} Wenzel–Cassie\;transition:W_{\mathrm{Roughness}}=W_{\mathrm{Roughness},\;c\;}\left(h_{c},w_{c},a_{c}\right)\\ Wenzel\;state:W_{\mathrm{Roughness}}>W_{\mathrm{Roughness},\;c\;}\left(<h_{c},\;or\;>w_{c},\;or\;<a_{c}\right)\\ Cassie\;\;state:W_{\mathrm{Roughness}}<W_{\mathrm{Roughness},\;c\;}\left(>h_{c},\;or\;<w_{c},\;or\;>a_{c}\right) \end{array}$$

Based on Equation (10), the critical wetting parameter may be expected during the Wenzel–Cassie transition, which is closely related to the geometric characteristic of surface roughness. Furthermore, the Wenzel state occurs when *W*_Roughness_ exceeds the critical value (*W*_Roughness_ > *W*_Roughness,c_), while the Cassie state is found when *W*_Roughness_ is below the critical value (*W*_Roughness_ < *W*_Roughness,c_). This understanding can be applied to elucidate the mechanism of the wetting transition from Wenzel to Cassie states and to design optimal superhydrophobic surfaces.

From the above discussion, to obtain the Cassie state, it is necessary to make the *W*_Roughness_ of surface roughness less than the critical *W*_Roughness,c_ (<*W*_Roughness,c_). According to Equation (10), the Cassie state may be expected as the pillar height being higher than *h*_c_ (>*h*_c_), or the separations between roughness being less than *w*_c_ (<*w*_c_), or the pillar size being larger than *a*_c_ (>*a*_c_). These may be utilized to understand the dependence of superhydrophobicity on surface roughness reported in previous MD simulations [34,35,37,38].

In this study, square pillars are considered, and *W*_Roughness_, as defined in Equation (9), is employed to investigate the effects of surface roughness on the Wenzel–Cassie transition. Surface roughness primarily influences the structure of interfacial water. Consequently, it is found that the wetting transition from Wenzel to Cassie states may be influenced not only by the size of the surface roughness but also by the geometric shape of the surface roughness. Furthermore, it is suggested that the Wenzel–Cassie transition may also be affected by the hierarchical structure of the solid surface, a phenomenon reported in many studies.

Additionally, based on Equation (9), it is also found that the critical parameter *W*_Roughness,c_ may be directly related to the Gibbs free energies of water, such as Δ*G*_Water-water_ and Δ*G*_DDAA_. It is well established that the structure of liquid water is influenced by temperature, pressure, and dissolved solutes. Therefore, the Wenzel–Cassie transition could also be influenced by temperature, pressure, dissolved solutes, and the molecular polarity of the solid surface. These factors can be used to modulate the wettability of superhydrophobic surfaces.

According to Equation (9), the wettability of a superhydrophobic surface is predicted to be independent of the size of the water droplet. This prediction is based on the assumption that the droplet size is much larger than the geometric characteristics of the surface roughness. This indicates that as the droplet size decreases, the wettability of the solid surface may become dependent on the size of the water droplet. Therefore, for a given surface roughness, the observed wettability may be dependent on the size of the water droplet.

Surface roughness primarily affects the hydrogen bonding of interfacial water. To assess the impact of surface roughness on water structure, a wetting parameter, *W*_Roughness_, is proposed, which depends on the geometric features of the roughness, including pillar size, width, and height. During the Wenzel–Cassie transition, a critical *W*_Roughness,c_ is expected, corresponding to a critical *a*_c_, *w*_c_, and *h*_c_. The Cassie state occurs when *W*_Roughness_ is less than *W*_Roughness,c_, which can be achieved by altering the surface geometry, such as increasing pillar size (>*a*_c_), decreasing width (<*w*_c_), or increasing height (>*h*_c_).

### 3.2. MD Simulations

In this work, a wetting parameter, *W*_Roughness_, is proposed and used to characterize the effects of surface roughness on wettability. During the wetting transition from Wenzel to Cassie states, a critical wetting parameter, *W*_Roughness,c_, is expected. To understand the relationship between superhydrophobicity and surface roughness, MD simulations are conducted. In the work, square pillars are designed on a graphite surface to simulate surface roughness (Table 1). The effects of surface roughness on superhydrophobicity are studied by varying the pillar side length, the groove width between pillars, and the pillar height. Additionally, a few MD simulations were conducted to explore the dependence of hydrophobicity on droplet size.

**Figure 3:**
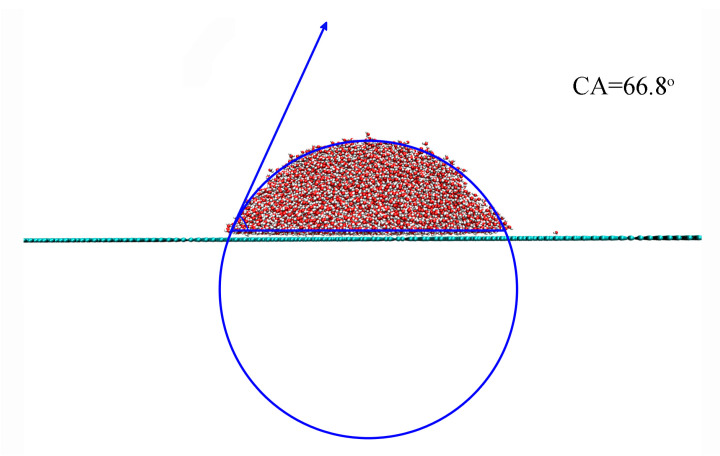
The measurement of contact angle (CA) on smooth surface.

**Figure 4:**
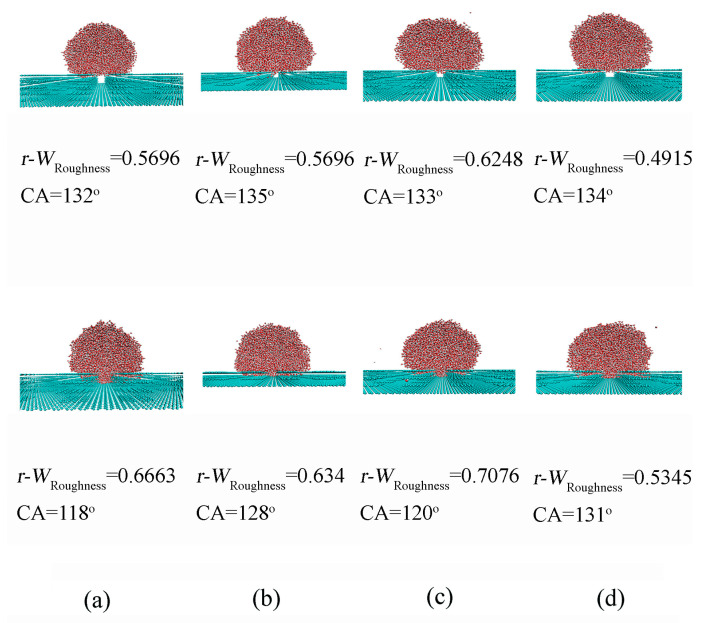
(**a**–**d**) The dependence of Wenzel–Cassie transition on surface roughness. The revised wetting parameters, *r-W*_Roughness_*,* are also shown. The geometric characteristics of surface roughness are shown in Table 1.

To investigate the effect of surface roughness on CA, the CA between a water droplet and a smooth graphite surface was first determined to be 66.8° (Figure 3). This value serves as a reference to evaluate the impact of surface roughness on wettability. It was found that the introduction of surface roughness led to an increase in CA from 66.8° to 139.7°, depending on the geometric characteristics of the roughness (Figure 4). Additionally, a sudden increase in CA was observed during the wetting transition from the Wenzel to Cassie states.

In the Wenzel state, the water droplet is fully wetted on the solid rough surface, with the grooves completely penetrated by the liquid phase. In contrast, in the Cassie state, the droplet is assumed to rest on the peaks of the roughness geometry, with the liquid not filling the grooves on the surface. During the transition from Wenzel to Cassie states, the bottom surface of the penetrated liquid gradually changes from conformal contact (complete penetration) to a final flat configuration (no penetration), which is described as a wetting transition from Wenzel to Cassie states (partial penetration) (Figure 4). The intermediate state can be used to characterize the Wenzel–Cassie wetting transition.

For a specific roughness with pillar size (*a*_x_ = 7.37 Å, *a*_y_ = 7.09 Å) and separation (*w*_x_ = 9.824 Å, *w*_y_ = 9.926 Å), the wettability shifts from Wenzel to Cassie states as the roughness height increases from 10.05 Å to 13.4 Å (Figure 4). This suggests the existence of a critical height *h*_c_ for the specific square roughness. Additionally, it was found that the critical height *h*_c_ is related to the pillar size and separations (Table 1). Therefore, based on the MD simulations, various critical values can be expected for different surface roughnesses. This indicates a close interdependence among the characteristics of surface roughness, *a*_x_, *a*_y_, *w*_x_, *w*_y_ and *h*, during the Wenzel–Cassie wetting transition. In this study, this interdependence is represented by the critical wetting parameter of roughness, *W*_Roughness,c_.

Based on the MD simulations, the *W*_Roughness_ parameter can be calculated for various surface roughness configurations. As the pillar height increases (or as the pillar size increases or the separations decrease), *W*_Roughness_ decreases (Table 1). Additionally, during the wetting transition from Wenzel to Cassie states, the critical wetting parameter, *W*_Roughness,c_, can be calculated to be in the range of 0.76–1.26, which is higher than the theoretical value of *W*_Roughness,c_ (0.47) at 293 K. This discrepancy may be closely related to the water model used in the MD simulations. In this study, the SPC/E water model is applied for water molecules. As noted by Chen et al. [69] and Vega et al. [70], the SPC/E model predicts a lower surface tension than observed in experimental measurements. Surface tension is closely related to Δ*G*_DDAA_ [44], which influences *W*_Roughness,c_. Therefore, the simulated results yield a higher *W*_Roughness,c_. Additionally, the molecular (or atom) size of surface roughness is neglected when Equation (9) is used to calculate the *W*_Roughness,c_. After the van der Waals radius of substrate is corrected, the revised *W*_Roughness_, *r*-*W*_Roughness_, is in the range of 0.49–0.71, which is close to the theoretical *W*_Roughness,c_ (Table 1).

In Ren et al.’s [38] MD simulations, a 4-site transferable intermolecular potential (TIP4P) model is used for water molecules. Based on their MD simulations on the wetting transition from Wenzel to Cassie states, the critical *W*_Roughness,c_ parameter is determined to be in the range of 0.65–1.05, and the corresponding revised *W*_Roughness,c_ lies in the range of 0.45–0.68 (Supplementary Materials).

Additionally, based on the above discussion, the Cassie states may be expected as the *W*_Roughness_ being less than *W*_Roughness,c_. This relationship can be used to understand the effects of surface roughness on hydrophobic states. In other words, the Cassie states is expected as the pillar height being higher than the critical height (=*a*_c_, =*w*_c_, >*h*_c_), or the separation being less than the critical width (=*a*_c_, <*w*_c_, =*h*_c_), or the size of pillar being larger than the critical size (>*a*_c_, =*w*_c_, =*h*_c_). These are consistent with the MD simulated results.

The OH vibration is closely related to the local hydrogen bonding of water [42,43], surface roughness primarily affects the structure of interfacial water. Therefore, the water can be divided into bulk water and interfacial water. According to MD simulations, the interfacial water layer exhibits a higher density compared to bulk water (Supplementary Materials). This observation is consistent with experimental measurements [71] and theoretical simulations [72] on confined water.

**Figure 5:**
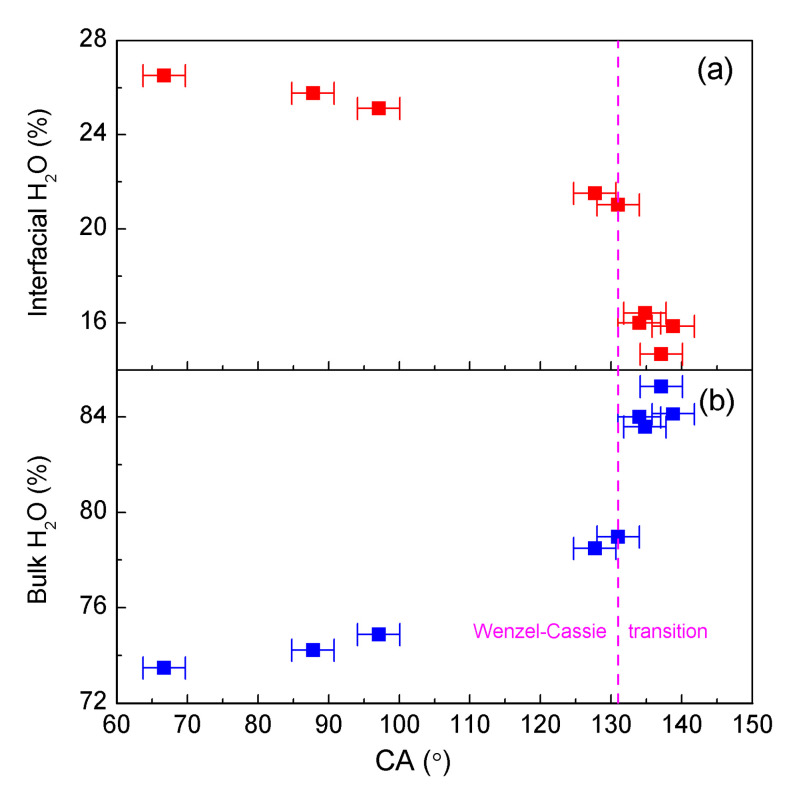
The dependence of interfacial (**a**) and bulk water (**b**) on CA.

The air–water and roughness–water interfaces mainly affect the structure of interfacial water. With increasing CA, this decreases the interfacial water and increases the bulk water (Figure 5). During the Wenzel–Cassie wetting transition, a notable decrease in interfacial water related to roughness accompanied by an increase in bulk water is observed (Figure 5). Therefore, the Wenzel–Cassie transition may be closely related to the transition between interfacial and bulk water.

**Figure 6:**
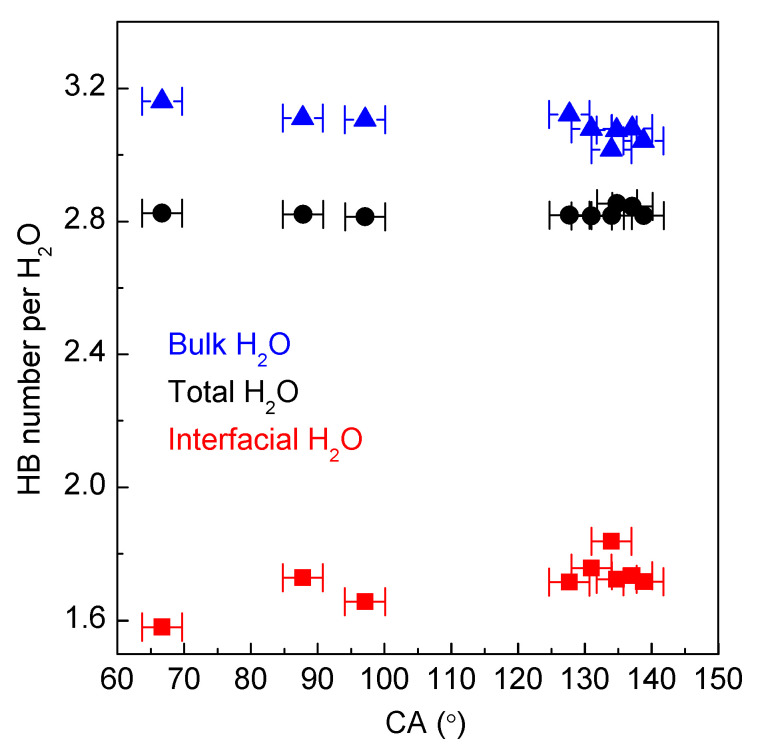
The changes of hydrogen bondings of interfacial and bulk water with CA.

From the MD simulations, the number of hydrogen bonds per water molecule may be determined. It is found that the hydrogen bonding of bulk water may be higher than that of interfacial water (Figure 6). Maximizing the hydrogen bonding of water leads to a decrease in interfacial water and an increase in bulk water. Therefore, the Wenzel–Cassie wetting transition can be ascribed to the structural competition between interfacial and bulk water. In combination with our recent studies [64,65], the Wenzel–Cassie transition may be closely related to hydrophobic interactions.

From this work, the Wenzel–Cassie wetting transition is closely related to the geometric characteristics of surface roughness, and Cassie state is expected for the substrate surface as the *W*_Roughness_ being less than *W*_Roughness,c_. This is assumed that the water droplet size may be significantly larger than the roughness features of the superhydrophobic surface. This indicates that the wettability may be related to the size of the water droplet. In comparison with the surface roughness, the wettability of smaller water droplets may be different from the larger water droplets.

**Figure 7:**
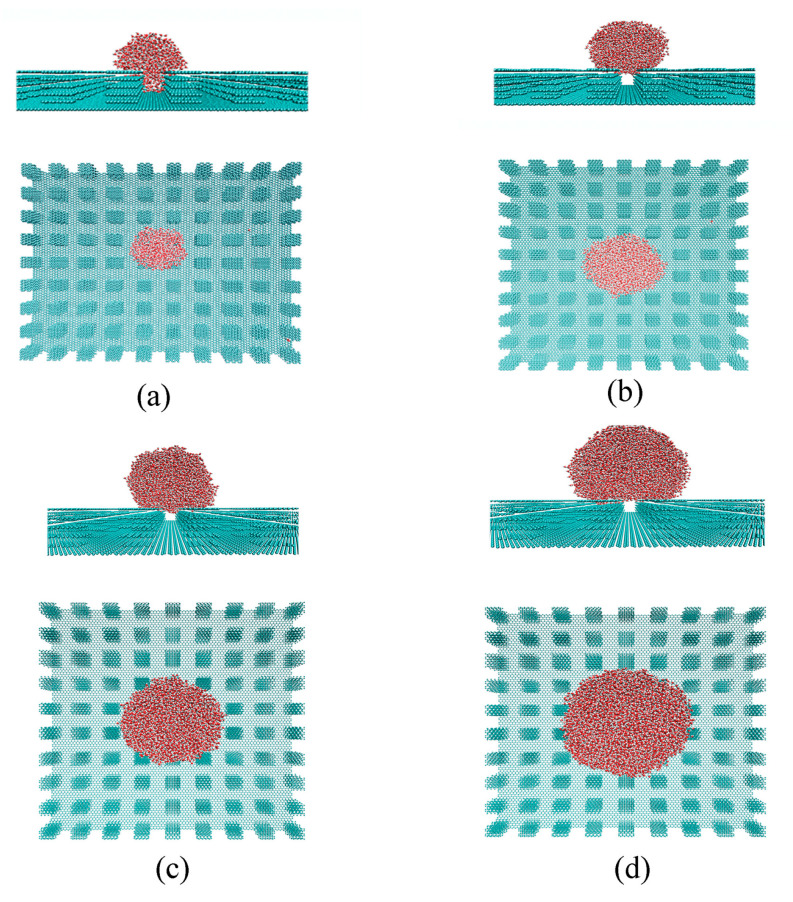
The dependence of wettability on water droplet size. Various water cubic box was initially placed on the solid surface (*a*_x_ = 9.824 Å, *a*_y_ = 8.508 Å, *w*_x_ = 12.28 Å, *w*_y_ = 12.05 Å, *h* = 16.75 Å), such as 30 Å × 30 Å × 30 Å (**a**), 40 Å × 40 Å × 40 Å (**b**), 50 Å × 50 Å × 50 Å (**c**), and 60 Å × 60 Å × 60 Å (**d**). Both side and top views of the systems are presented after thermodynamic equilibrium was reached.

In this study, the dependence of superhydrophobicity on droplet size was investigated by placing initial liquid phases of various cubic sizes on the solid surface, specifically 30 Å × 30 Å × 30 Å, 40 Å × 40 Å × 40 Å, and 50 Å × 50 Å × 50 Å. From the MD simulations, it was observed that increasing the size of the water droplet resulted in a wetting transition from the Wenzel to the Cassie state (Figure 7). For the specific substrate studied, the Wenzel–Cassie wetting transition was observed when the droplet diameter reached approximately 75 Å, corresponding to an initial water box of 40 Å × 40 Å × 40 Å. Therefore, the Cassie state is expected for droplets larger than 75 Å, where the droplet size significantly exceeds the geometric features of the surface roughness.

In addition, based on the thermodynamic analysis of the Wenzel–Cassie transition, it is derived that superhydrophobicity may also be influenced by temperature, pressure, the molecular polarity of the solid surface, the geometric shape of surface roughness, and the hierarchical structure of the solid surface. Further study is necessary.

## 4. Conclusions

Superhydrophobicity is primarily influenced by the geometric characteristics of surface roughness. Building upon our structural studies on water and the air–water interface, a *W*_Roughness_ parameter, closely related to the geometric characteristics of the roughness, is proposed and applied to investigate the wetting transition from Wenzel to Cassie states on hydrophobic surfaces. During the Wenzel–Cassie wetting transition, a critical *W*_Roughness,c_ is expected, which may be characterized by the critical geometric characteristics of surface roughness, such as *a*_c_, *w*_c_, and *h*_c_. Based on thermodynamic analysis, the Cassie state is favored for a hydrophobic surface when the *W*_Roughness_ is less than the critical *W*_Roughness,c_ (*W*_Roughness_ < *W*_Roughness,c_). This can be achieved by increasing the pillar size (>*a*_c_), decreasing the width (<*w*_c_), or increasing the height (>*h*_c_). This approach can be used to modulate the wetting transition between the Wenzel and Cassie states.

## Figures and Tables

**Table 1:** Simulated systems. During the simulations, square pillars are designed on a graphite surface to simulate surface roughness. The wetting parameter (*W*_Roughness_) and revised *W*_Roughness_ (*r-W*_Roughness_) are also shown.

System	*a*_x_ (Å)	*a*_y_ (Å)	*w*_x_ (Å)	*w*_y_ (Å)	*h* (Å)	Wettability	*W*_Roughness_	*r-W*_Roughness_	CA
a	7.37	7.09	7.37	7.399	10.05	Cassie	0.9713	0.5696	132°
	7.37	7.09	7.37	7.399	6.7	Wenzel	1.1803	0.6663	118°
b	7.37	7.09	9.824	9.926	13.4	Cassie	0.9713	0.5696	135°
	7.37	7.09	9.824	9.926	10.05	Wenzel	1.1106	0.634	128°
c	7.37	7.09	12.28	12.05	13.4	Cassie	1.0906	0.6248	133°
	7.37	7.09	12.28	12.05	10.05	Wenzel	1.2696	0.7076	120°
d	9.824	8.508	12.28	12.05	16.75	Cassie	0.7632	0.4915	134°
	9.824	8.508	12.28	12.05	13.4	Wenzel	0.8444	0.5345	131°

## Data Availability

The original contributions presented in this study are included in the article/Supplementary Material. Further inquiries can be directed to the corresponding author.

## Supplementary Materials

The following supporting information can be downloaded at: https://www.mdpi.com/article/10.3390/ma18030543/s1, Figure S1: Density distributions in Wenzel and Cassie states along y–z plane; Table S1: Based on Ren et al.’s [38] MD simulations on Wenzel–Cassie transition, the *W*_Roughness_ and *r-W*_Roughness_ are determined.
