# Supplementary material for: Wetting Transition from Wenzel to Cassie States: Thermodynamic Analysis
Source: Materials (Basel). 2025 Jan 24;18(3):543. doi: 10.3390/ma18030543 (PMC11818709; doi:10.3390/ma18030543)
Supplement: Supplementary file 1 [file materials-18-00543-s001.zip › materials-3406066-supplementary.pdf]

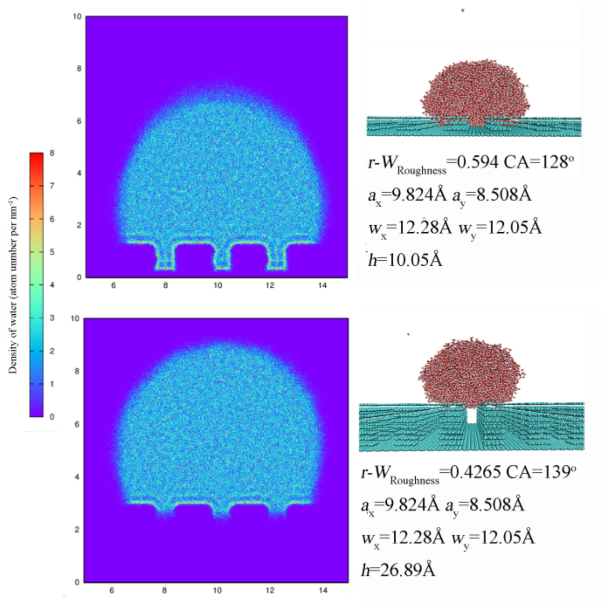

Figure S1. Density distributions in Wenzel and Cassie state along y-z plane.

Table S1. Based on Ren et al. [38] MD simulations on Wenzel-Cassie transition, the $W_{Roughness}$ and r-$W_{Roughnesr}$ are determined.

a_x (Å)	a_y (Å)	w_x (Å)	w_y (Å)	h (Å)	wettability	$W_{Roughness}$	r-$W_{Roughness}$
11.36	11.07	22.73	20.91	20.1	Cassie	0.7880	0.5277
11.36	11.07	22.73	20.91	16.75	Wenzel	0.8743	0.5785
11.36	11.07	14.2	13.53	13.4	Cassie	0.7299	0.4934
11.36	11.07	14.2	13.53	10.05	Wenzel	0.8542	0.5667
11.36	11.07	9.94	8.61	10.05	Cassie	0.6884	0.469
11.36	11.07	9.94	8.61	6.7	Wenzel	0.8542	0.5667
11.36	11.07	5.68	6.15	6.7	Cassie	0.7050	0.4788
11.36	11.07	5.68	6.15	3.35	Wenzel	1.0532	0.6838
11.36	11.07	5.68	3.69	6.7	Cassie	0.6552	0.4495
11.36	11.07	5.68	3.69	3.35	Wenzel	0.9537	0.6252
